# Preparation of eGaIn NDs/TPU Composites for X-ray Radiation Shielding Based on Electrostatic Spinning Technology

**DOI:** 10.3390/ma17020272

**Published:** 2024-01-05

**Authors:** Jing Wang, Kaijun Wang, Jiale Wu, Jin Hu, Jiangfeng Mou, Lian Li, Yongjin Feng, Zhongshan Deng

**Affiliations:** 1 College of Materials Science and Engineering, Kunming University of Science and Technology, 121 Street, Wenchang Road 68, Kunming 650093, China; 18886018439@163.com (J.W.); 916918wm@sina.com (J.W.); 18786854957@163.com (J.M.); lllian6789@163.com (L.L.); 2 Southwestern Institute of Physics, Huangjing Road 5, Chengdu 610041, China; fengyj@swip.ac.cn; 3 Beijing Key Laboratory of Cryo-Biomedical Engineering, Technical Institute of Physics and Chemistry, Chinese Academy of Sciences, Beijing 100190, China; zsdeng@mail.ipc.ac.cn

**Keywords:** electrospinning, liquid metals eGaIn alloys, polymer composite, low-energy X-ray shielding, Phy-X/PSD, WinXCom

## Abstract

Thermoplastic polyurethane (TPU) composites with eutectic gallium (Ga) and indium (In) (eGaIn) fillings of 0 wt%–75 wt% were prepared using the electrostatic spinning method. Field emission scanning electron microscopy (SEM), X-ray diffraction (XRD), and Fourier-transform infrared (FTIR) spectroscopy were used to characterize the eGaIn NDs/TPU composites. To evaluate their X-ray shielding properties, Phy-X/PSD and WinXCom were employed to calculate the mass attenuation coefficients, linear attenuation coefficients, half-value layers, tenth value layers, mean free paths, and adequate atomic numbers of the eGaIn NDs/TPU composites. The SEM results indicated that the eGaIn nanodroplets were evenly distributed throughout the TPU fibers, and the flowable eGaIn was well-suited for interfacial compatibility with the TPU. A comparison of the eGaIn NDs/TPU composites with different content levels showed that the composite with 75 wt% eGaIn had the highest *μ_m_* at all the evaluated energies, indicating a superior ability to attenuate X-rays. This non-toxic, lightweight, and flexible composite is a potential material for shielding against medical diagnostic X-rays.

## 1. Introduction

X-rays benefit medical diagnosis, cancer treatment, nuclear medicine imaging and therapy, and nuclear science research [1,2]. However, within acceptable exposure limits, X-rays can cause irreversible damage to human cells and tissues and severely affect the immune, reproductive, and central nervous systems if radiation shielding is inadequate [3,4,5]. The traditional radiation shielding material is primarily lead (Pb); the high atomic number and density of Pb give it a high probability of interacting with photons, resulting in good X-ray shielding properties [6]. However, owing to the toxicity of Pb, which can endanger the user’s health, it is crucial to replace lead with non-toxic materials [7]. Consequently, many Pb-free materials have been developed, including tungsten carbide, Mg–Gd–Y–Zn–Zr, Bi–Sn–Zn, and TeO_2_–ZnO–Na_2_O–Ag_2_O [8,9,10,11]. Nevertheless, alloys are stiff and bulky, whereas glass is fragile and prone to discoloration [12]. Therefore, the use of such materials in wearable radiation-protective clothing is limited.

Shielding composites have become popular materials for radiation shielding applications in recent years. These composites use polymers as the matrix and Pb-free materials as fillers because of their light weight, soft properties, high mechanical strength, excellent ease of processing and stability, and low maintenance cost [13]. Seulgi Kim, Yun Hee Ahn, and colleagues synthesized tungsten (W) nanoparticles modified with a blend of boron nitride nanosheets (BNNS) and combined with polyethylene (PE) to create W-BNNS/PE composites for shielding [14]. In addition, Haibin Wang et al. prepared Gd_2_O_3_/polyether ether ketone (PEEK) composites using Gd_2_O_3_ as a filler for X-ray shielding applications [15]. These polymer composites are suitable candidates for X-ray shielding.

Electrostatic spinning technology can control the diameter of polymer fibers from the nanoscale to the microscale, and the spun nanofiber mats have high porosity, small diameters, and good mechanical properties [16,17,18]. This technique is becoming an effective method for preparing new X-ray shielding materials. Nurul, Wan, et al. prepared n–ZnO/n–Bi_2_O_3_/epoxy resin-PVA X-ray energy shielding mats using electrostatic spinning [19]. Furthermore, Munirah et al. prepared PVA/epoxy–PVA X-ray energy shielding mats with the good X-ray shielding properties of PVA/Bi_2_O_3_ and PVA/WO_3_ nanofiber mats [20]. Unfortunately, agglomeration between particles is unavoidable due to excessive additions.

In addition, Ye et al. combined spraying with direct nanosecond ultraviolet (UV) laser sintering to rapidly fabricate highly conductive, micron-thick flexible films of sintered liquid metal submicron particles [21]. Liu et al. used eutectic gallium (Ga) and indium (In) (eGaIn) liquid metals to prepare a polymeric soft conductor that can be repeatedly stretched [22]. Deng et al. produced a flexible X-ray shielding film by laminating a silicon film with low-temperature Ga_61_In_25_Sn_13_Zn_1_ liquid metal [23]. Moreover, medical diagnostic X-rays typically have energies below 100 keV, falling within the weak absorption range of Pb [24]. However, Ga and In, low-melting-point metals, considerably improve X-ray absorption in the photon energy range between 0 and 80 keV [25]. These findings show that liquid alloys with flexibility overcome the rigidity of conventional alloys and that the flexibility, machinability, and good X-ray shielding properties of liquid alloys provide a broader range of applications, and the liquid metal can be well bonded to polymers. Therefore, this study aims to prepare polymeric nanocomposites used as X-ray shielding materials by dispersing eGaIn nanodroplets (NDs) of different weight percentages (wt%) in thermoplastic polyurethane (TPU) using the electrostatic spinning method.

## 2. Material and Method

### 2.1. Material

The materials used were TPU powder (Badische Anilin-und-Soda-Fabrik, Ludwigshafen, Germany) with a density of 1.2 g/cm^3^, Ga metal (China Lead) with a density of 5.91 g/cm^3^, In (Guangzhou Metallurgical Company, Guangzhou, China) with a density of 7.31 g/cm^3^, and polyvinylpyrrolidone (PVP) powder with a molecular weight (Mw) of 89,000–98,000 g/mol and density of 1.3 g/cm^3^. eGaIn is produced as liquid metal (LM) with a melting point of 14.7 °C by alloying 75 wt% Ga and 25 wt% In. The raw Ga and In of 99.99% purity are weighed in the corresponding weight ratios and then heated with stirring at 60 °C for 2 h.

### 2.2. Preparation of eGaIn NDs/TPU Nanofiber Mats

**Figure 1:**
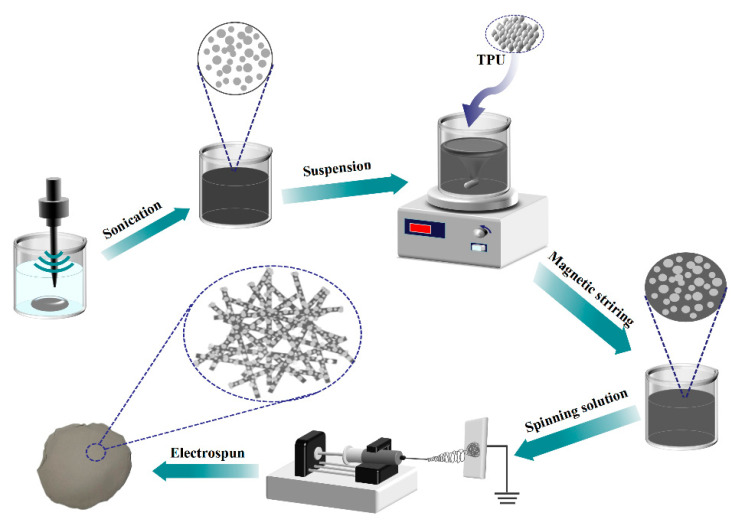
Schematic of the preparation procedure for eGaIn NDs/TPU shielding radiation composites.

Figure 1 shows the preparation process for eGaIn NDs/TPU nanofiber mats. First, eGaIn was dispersed in N, N-dimethylformamide (DMF) containing PVP, and the eGaIn NDs–DMF emulsion was obtained by ultrasonic dispersion for 5 h. Subsequently, to obtain the electrostatic spinning solution, TPU was dissolved in the emulsion and magnetically stirred for 12 h. The electrostatic spinning solution was then loaded into a 10 mL syringe with a 21-gauge needle and electrospun at a flow rate of 0.5 mL·h^−1^. A voltage of 15 kV was applied between the needle and the copper foil collector, which were located 15 cm from each other. The spun-bonded films were dried in a vacuum drying oven at 45 °C for 2 h to prepare the eGaIn ND/TPU. The listed solutions in Table 1 were used for the preparation.

### 2.3. Material Characterization

Field emission scanning electron microscopy (SEM; EOL JSM-7800F, Tokyo, Japan) was used to observe the micromorphology of the samples. Energy dispersive spectroscopy (EDS) was used to observe the elemental distributions. Furthermore, X-ray diffraction (XRD, Cu Kα, Tokyo, Japan) was used to characterize the crystal structure and phase composition of the samples. The eGaIn NDs/TPU composites were analyzed via Fourier-transform infrared (FTIR) spectroscopy with a Thermo Scientific Nicolet iS20 (Waltham, MA, USA) spectrometer in the reflection mode set at 4000–400 cm^−1^. The density was determined using the Archimedes drainage method, and the average of three measurements was taken. The viscosity was measured employing a digital rotational viscometer (NDJ-8S, Dobetter Group of Corporation, Shanghai, China).

### 2.4. Evaluation of X-ray Radiation Shielding Performance

Phy-X/PSD (https://phy-x.net/PSD), an online software program, calculates essential parameters related to photon shielding and dosimetry, such as linear attenuation coefficients (LAC), mass attenuation coefficients (MAC), half-value layers (HVL), tenth value layers (TVL), mean free paths (MFP), and adequate atomic numbers (Zeff). The WinXCom software (https://physics.nist.gov/PhysRefData/Xcom/html/xcom1.html), developed by Berger et al., calculates scattering, photoabsorption, pairwise photon cross-sections, and total attenuation coefficients between 1 keV and 100 GeV. A photon cross-section library was used through the WinXCom software to obtain the MAC for each element. The MAC evaluated by WinXCom was compared to those calculated by Phy-X/PSD to demonstrate the dependability of the Phy-X/PSD measurements.

### 2.5. Basic Photon Attenuation Parameters

The significant parameters related to shielding are the MAC, LAC, HVL, TVL, MFP, and Z_eff_. MAC (*µ_m_*) represents the interaction probability per unit volume in the shield material [26].

(1) $$\mu_{m}=\left(\frac{\mu }{\rho }\right)=\sum_{i}w_{i}{\left(\frac{\mu }{\rho }\right)}_{i}$$

The μ represents the LAC, and *ρ* is the density of the alloy. ${\left(\frac{\mu }{\rho }\right)}_{i}$ denotes the $\mu_{m}$ of each element in the alloy, and $w_{i}$ is the element weight fraction.

Another crucial metric for evaluating material attenuation performance is the LAC (μ), which describes the fraction of X-rays or gamma rays absorbed or scattered per unit thickness of the absorber. This value considers the number of atoms in a cubic centimeter of material and the probability of a photon being scattered or absorbed by an atomic nucleus or electron.

(2) $$\mu =\mu_{m}\rho$$

The HVL describes the thickness of the material required to reduce the energy of the original photon by 50%, the TVL denotes the thickness of the material required to reduce the energy of the original photon by 10%, and the MFP determines the average path between two interacting photons. The following formulas show the relationship between these parameters and the line attenuation factor (μ) [27].

(3) $$HVL=\frac{In2}{\mu }$$

(4) $$TVL=\frac{In10}{\mu }$$

(5) $$MFP=\frac{1}{\mu }$$

The *Z_eff_* varies with energy, as is required for pure elemental atoms with unique atomic numbers, and describes the composition of the material based on the equivalent element [28].

(6) $$Z_{eff}=\frac{\sigma_{a}}{\sigma_{e}}$$

where $\sigma_{a}$ is the total of the atomic cross-sections of the alloy and $\sigma_{e}$ is the total of the electronic cross-sections of the alloy. $\sigma_{a}$ can be obtained from $\mu_{m}$ using the below equation [29]:

(7) $$\sigma_{a}=\frac{N\mu_{m}}{N_{A}}$$

where $N_{A}$ is the Avogadro constant and *N* is the total number of atoms.

The $\sigma_{e}$ for each sample can be evaluated with the following equation:

(8) $$\sigma_{e}=\frac{1}{N_{A}}\left(\sum_{i}\frac{f_{i}A_{i}}{z_{i}}{\left(\mu_{m}\right)}_{i}\right)$$

where $z_{i}$ is the atomic number, $f_{i}$ is the molar fraction, and $A_{i}$ is the atomic weight of its group element.

## 3. Results and Discussion

### 3.1. eGaIn NDs Structural and Morphological Analysis

**Figure 2:**
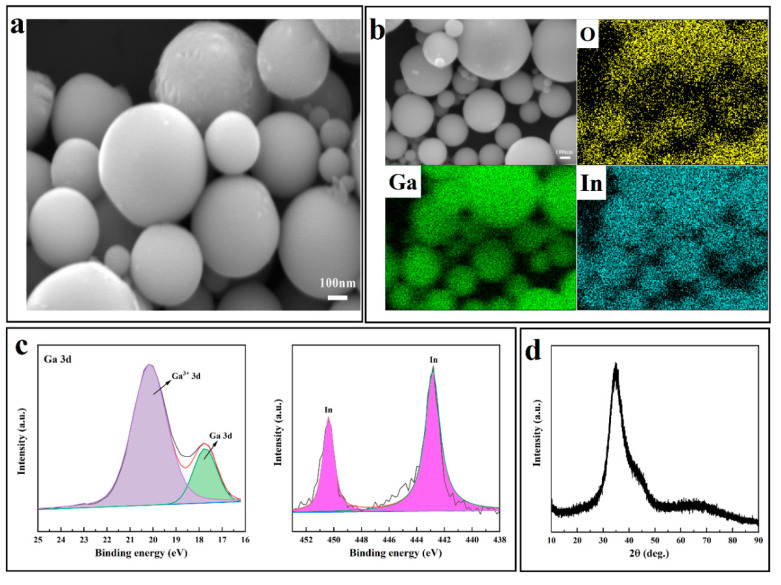
(**a**) SEM image of eGaIn NDs. (**b**) elemental mappings. (**c**) XPS spectra of eGaIn NDs. (**d**) XRD patterns of eGaIn NDs.

The ultrasonic treatment of eGaIn in the DMF solution disperses the eGaIn into nanodroplets, in which DMF and PVP can prevent the agglomeration of eGaIn NDs, and uniform eGaIn NDs–DMF emulsions can be obtained. Figure 2a shows the SEM image of the eGaIn NDs. The ultrasonic treatment breaks up the eGaIn and separates it into nanodroplets because of the large oscillating shear force generated during the ultrasonic treatment. The EDS image in Figure 2b shows the distribution of the three elements O, Ga, and In in eGaIn NDs. In addition, the O element was caused by the Ga_2_O_3_ film formed on the surface of eGaIn NDs during the ultrasonic process, and this oxide layer functioned as a protective shell for eGaIn NDs, maintaining the transient structural stability of the spherical eGaIn NDs and ensuring that these nanoscale droplets remained mechanically robust and prevented agglomeration [30]. Furthermore, the presence of two chemical states (metal and oxidation stoichiometry) of metallic Ga observed in the XPS feature spectra, as shown in Figure 2c, indicates that Ga is slightly oxidized to Ga_2_O_3_ during sonication. Since Ga^3+^ readily combines with −OH and −COOH bonds to form GaOOH, large amounts of Ga^3+^ are essential for bonding the eGaIn droplets to TPU [31]. The bun peaks at 2*θ* = 30°–40° indicate that the prepared eGaIn alloys are amorphous in Figure 2d.

### 3.2. eGaIn NDs/TPU Nanofiber Mats: Structural and Morphological Analysis

**Figure 3:**
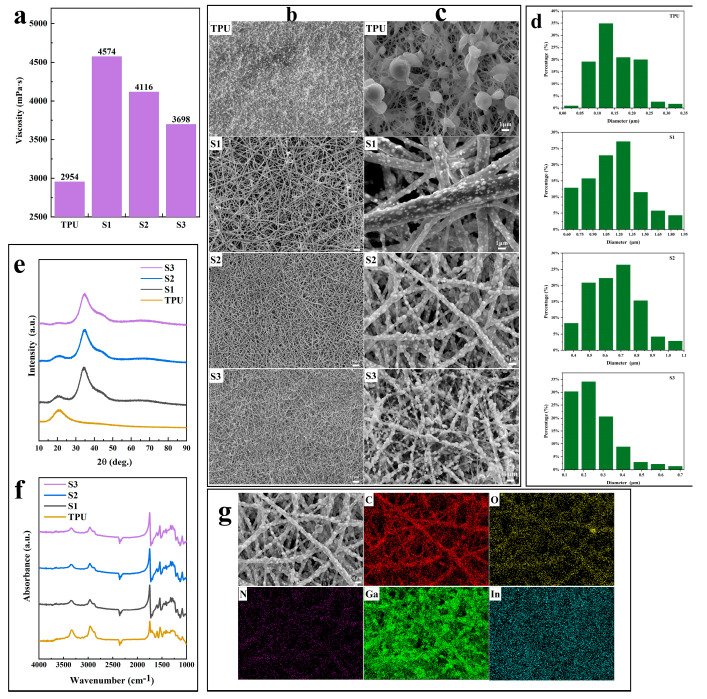
eGaIn NDs/TPU composite. (**a**) Viscosity of spinning solutions of eGaIn NDs/TPU at different concentrations; (**b**,**c**) SEM pictures of eGaIn NDs/TPUs; (**d**) Fiber diameter distribution map; (**e**) XRD plots of eGaIn NDs/TPU complexes; (**f**) FTIR spectra of eGaIn NDs/TPU composites; (**g**) Elemental mappings of 66.7 wt% eGaIn NDs s/TPU composite.

In the electrospinning process, the charged spinning solution is expelled from the droplets under an electric field, forming fibers [32]. The changes in the viscosity of the spinning solution significantly influence the electrospun fibers. The applied electric field fails to stabilize the charged jets for low viscous polymer solutions, creating Rayleigh instabilities that break the polymer chains into particles before they reach the receiver board. These fragments generate bead-like nanofibers. As the concentration of the polymer solution increases, so does the viscosity of the solution. These entangled chains overcome the surface tension, resulting in uniform, sphere-free electrospun nanofibers [33,34]. Thus, viscosity is crucial for obtaining continuous and homogeneous fibers. Figure 3a shows the viscosity of eGaIn NDs/TPU spinning solutions with eGaIn content between 0 wt% and 75 wt%.

The viscosity of the spinning solution is at its lowest without eGaIn addition, increases when 50 wt% of eGaIn is added, and then decreases as the eGaIn content increases. This result might be similar to the different viscosities of the oil–water mixture before and after the phase transition. The transition phase is a continuous oil or water phase in a liquid mixture that becomes a discrete internal phase under specific conditions, and the original discrete internal phase becomes a new continuous external phase [35]. The viscosity of eGaIn is lower than that of the TPU solution (the viscosity of eGaIn (1.99 mPa·s) is similar to that of water (2.98 mPa·s)) [36]. When 50 wt% of eGaIn was added to the TPU solution, the eGaIn remained a discrete inner phase, increasing the viscosity of the entire system. With the increase in eGaIn, the original eGaIn discrete phase gradually became a continuous outer phase, and the viscosity of the system decreased. Figure 3b,c show the SEM images of the electrospun eGaIn NDs/TPU fiber mats with eGaIn nanoloading from 0 wt% to 75 wt%. The SEM shows that the electrospun TPU nanofibers appear as many bead-like fibers compared with the samples containing fillers. The pores of other eGaIn NDs/TPU fiber mats decrease with increasing eGaIn content, and the density between the fibers increases. When the eGaIn content was 50 wt% and 66.7 wt%, the eGaIn NDs were uniformly embedded on the surface or inside the nanofibers, respectively. When the eGaIn content was 75 wt%, the eGaIn NDs were mainly loaded on the fibers as agglomerates, and a small amount of bead-like fibers appeared. Figure 3d shows the fiber diameter distributions. The fiber diameter distributions show that the electrospun TPU nanofibers have the lowest average diameters compared to the filled samples. Most of these fibers are 25–35 nm. The other fiber diameters decreased with the increase in the eGaIn content. The major fiber diameter distributions of eGaIn NDs/TPU fiber mats loaded with 50 wt%, 66.7 wt%, and 75 wt% were 0.6–1.95 μm, 0.4–1.1 μm, and 0.1–0.7 μm, respectively.

Figure 3e shows the XRD patterns of the TPU nanofiber and eGaIn NDs/TPU fiber mats. This XRD result shows a bun-shaped diffraction peak at 2*θ* = 20°, proving the presence of amorphous TPU. A broad peak at 2*θ* = 30°–40° corresponds to an eGaIn alloy with an amorphous phase, indicating that eGaIn nanoparticles are still liquid in the nanofibers. In addition, the XRD peak intensity depends on the phase content of the sample. With the gradual increase in the eGaIn content and the gradual decrease in the TPU content in the composites, the bun-like peak gradually disappeared, i.e., the TPU diffraction peak intensity decreased. However, the eGaIn peak intensity did not change significantly. Figure 3f analyzes the FTIR spectra of the eGaIn NDs/TPU nanofiber mats. The C–O stretching vibration of the TPU is at approximately 1731 cm^−1^, the N–H bending vibration at 1539 cm^−1^, and the C–N stretching vibration at 1322 cm^−1^ [37]. However, the FTIR spectroscopy of all samples showed only the characteristic peaks of the TPU and no peaks of new functional groups, meaning that even if the eGaIn NDs were filled, the internal structure of the TPU would not be changed. No chemical reaction would occur between the eGaIn NDs and the TPU. Furthermore, sample 2 was selected as a representative to characterize the microdistribution of the eGaIn NDs in the fibers. In Figure 3g, Ga and In are evenly spread throughout the fibers, indicating that eGaIn NDs are evenly spread in the TPU, ensuring fundamental stability in the radiation shielding characteristics of the composites.

### 3.3. Evaluation of X-ray Shielding Performance of eGaIn NDs/TPU Nanofiber Mats

**Figure 4:**
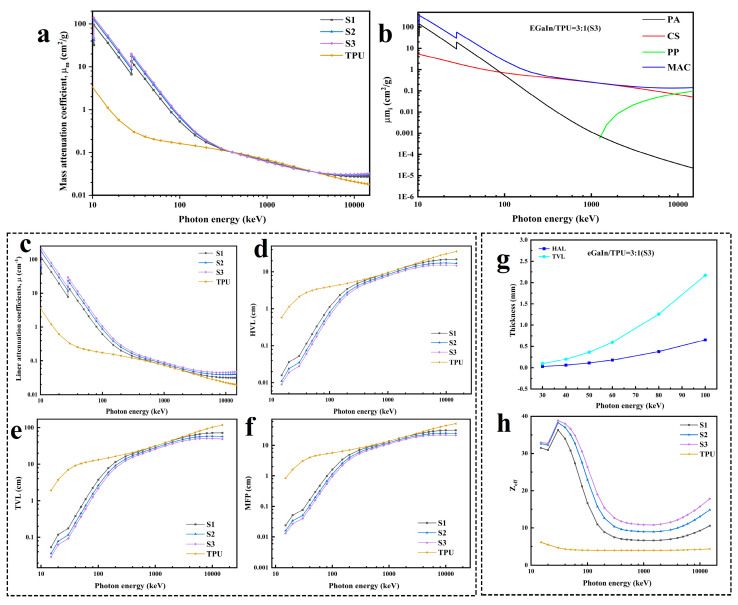
(**a**) MAC of the composites; (**b**) PA, CS, and PP contributions to the ${\mu_{m}}_{i}$ Sample 3; (**c**–**f**) μ, HVL, TVL, and MFP values of the eGaIn NDs/TPU composites; (**g**) HVL and TVL of Sample 3; and (**h**) Z_eff_ of the composites.

Table 2 shows the variation of the MAC of the eGaIn NDs/TPU nanofiber mats with a photon energy of 15–15,000 keV. The $\mu_{m}$ values obtained from the Phy-X theoretical calculations and the WinXCom code are matched to determine the accuracy of the data obtained with the Phy-X/PSD online software. As shown in Table 2, the Phy-X and XCom calculation results correlate well, verifying the correctness of Phy-X. The radiation shielding principle of radiation protection materials is based on attenuation. The effect of waves or rays is reduced by blocking or bouncing particles through the blocking material. The theory of secondary effects, comprising the photoelectric, Compton, and electron pair effects, elucidates the interaction between the material and the photons. The incident X-rays excite electrons in atoms in the material, and the excited electrons produce secondary radiation and photoelectrons that convert the energy of the incident rays, causing attenuation. However, since the energy of X-rays is 100 eV–10 MeV, the energy absorption of X-rays is mainly dominated by the photoelectric effect and the Compton effect in this relatively low energy range. Figure 4a shows that the MAC value of 10–15,000 keV is highly dependent on the composition and photon energy of the sample. A general decreasing trend exists in the MAC values of the eGaIn NDs/TPU nanofiber mats. In particular, in the 10–500 keV range, the MAC value declines precipitously with the rising photon energy because, in this energy range, incoming photons will elastically impact orbital electrons outside the nucleus, and the photons will interact with the electrons to transfer all of the energy to the electrons, thereby dissipating the energy of the incident photons. This process is the photoelectric absorption that predominates in the low-energy area and produces an inverse relationship between MAC values and energy levels [38].

Meanwhile, the mass absorption coefficient of the eGaIn NDs/TPU nanofiber mats clearly increases with increasing eGaIn NDs because the photoelectric cross-section varies in proportion to Z^4–5^(Z refers to the atomic number) and in inverse proportion to the incident photon energy E^3.5^(E refers to the photon energy) [39]. Except for the pure TPUs, the MAC curves significantly increase at specific positions at 10–30 keV because of the significant photoelectric absorption at the specific absorption edges of the metallic elements of the eGaIn NDs/TPU nanofiber mats. The K-absorption edge for Ga is at 103.7 keV and for In at 279.4 keV, with peaks observed at both energy levels.

The radiation shielding mechanism of the eGaIn NDs/TPUs is explored using sample 3 (Figure 4b). Photoelectric absorption (PA) dominates in the low-energy range from 0 to 150 keV. The MAC sharply declines with rising photon energy because the PA cross-section is positively correlated with Z^4–5^ and inversely correlated with E^3.5^. The probability of the compton scattering (CS) effect gradually increases with increasing photon energy and occurs in the intermediate energy range of 50–10,000 keV. Since the CS cross-section positively correlates with Z and E^−1^, the MAC curve approximates a smooth falling line at this point. When the photon energy exceeds 10,000 keV, the probability of the CS effect decreases while that of the pair production (PP) effect increases, and the photoelectric absorption dominates the interaction of high-energy photons with matter. Since the photoelectric production cross-section depends on Z^2^ and log(E), the MAC curve increases steadily as the energy of the photon rises [40].

The weakening of X-rays is the process by which X-rays interact with an object and produce a loss of energy as they penetrate the object. The degree of attenuation is valued as an LAC. As shown in Figure 4c, the higher the eGaIn loading in the eGaIn NDs/TPU nanofiber mats is, the higher the LAC is; the X-ray attenuation performance of sample 3 is the best. While ensuring that the shielding material can attenuate X-rays, the appropriate thickness determines the effective radiation shielding design. Figure 4d,e show the HVL and TVL graphs, where the values of both parameters decrease with the eGaIn content, whereas the overall trend increases with the photon energy. Since the energy range of medical X-rays is 30–100 keV, sample 3 was chosen to analyze the HVL and TVL in this photon energy range. The thickness of the eGaIn NDs/TPU nanofiber mats required to attenuate the incident X-rays to 50% and 10% at a photon energy of 60 keV was chosen to be 0.179 cm and 0.595 cm (Figure 4g). Figure 4f shows that the MFP value decreases with increasing eGaIn content and increases with increasing photon energy. The smaller MFP indicates that the collision path between the photons is shorter and that the energy is attenuated more quickly. Therefore, sample 3 is the most suitable material for X-ray shielding.

Figure 4h shows the Z_eff_ variation of the eGaIn NDs/TPU nanofiber mats at 15–15,000 keV. The LM-free TPU has the lowest effective atomic number value, similar to a direct line. Simultaneously, the Z_eff_ value of the eGaIn NDs/TPU nanofiber mats rises with the increase in eGaIn NDs loading. In the energy range of 15–30 keV, an abrupt increase in the Z_eff_ value was observed owing to the K-absorption edge at 30 keV. At 30–2000 keV, the Compton scattering effect dominates in this region, and the CS cross-section is linear with Z and E, decreasing the Z_eff_ value with the increasing photon energy when a minimum Z_eff_ value is observed. When the photon energy is greater than 2000 keV, the interaction between the photon and matter is predominantly an electron pair production effect, the cross-section of which is correlated with Z^2^ and logE, and the generation of electron pairs slightly increases the Z_eff_ value.

## 4. Conclusions

Electrospun eGaIn NDs/TPU nanofiber mats with fillers ranging from 0 wt% to 75 wt% have been successfully prepared. These mats can be good candidates for Pb-free polymer-based materials as potential X-ray shielding materials because of their reasonably good X-ray attenuation capability. Furthermore, preparing eGaIn materials as X-ray shield fillers using the electrostatic spinning process produces lighter and more flexible materials than conventional Pb-based materials. In this study, the highest X-ray attenuation capability was achieved with 75 wt% eGaIn NDs/TPU nanofiber mats. Therefore, it is the most likely candidate for X-ray shielding material when preparing eGaIn NDs/TPU composites via electrostatic spinning.

## Figures and Tables

**Table 1:** List of prepared electrospun solutions with different weight percentages (wt%) of eGaIn and TPU.

Sample ID	eGaIn NDs/TPU	eGaIn NDs Loading (wt%)	Elemental Composition (wt%)						ρ (g/cm^3^)
			Ga	In	C	H	O	N	
S1	1/1	50.0	32.8	17.2	29.6	3.3	14.6	2.5	1.168
S2	2/1	66.7	43.4	23.4	19.6	2.2	9.7	1.7	1.334
S3	3/1	75.0	48.9	26.0	14.8	1.7	7.3	1.3	1.487
TPU	0/1	0.0	0	0	59.1	6.6	29.1	5.2	1.082

**Table 2:** MAC (cm^2^/g) of the eGaIn NDs/TPU composites using the Phy-X and WinXCom procedures and relative standard error.

Photon Energy (keV)	S3			S2		
	Phy-X (cm^2^/g)	WinXCom (cm^2^/g)	Δ (%)	Phy-X (cm^2^/g)	WinXCom (cm^2^/g)	Δ (%)
15	53.5957	53.6000	−0.0043	47.7775	47.7800	−0.0025
20	24.6719	24.6700	0.0019	22.0004	22.0000	0.0004
30	16.6127	16.6100	0.0027	14.8893	14.8900	−0.0007
40	7.7309	7.7320	−0.0011	6.9403	6.9400	0.0003
50	4.2364	4.2370	−0.0006	3.8119	3.8120	−0.0001
60	2.6020	2.6020	0.0000	2.3482	2.3480	0.0002
80	1.2310	1.2310	0.0000	1.1199	1.1200	−0.0001
100	0.7135	0.7135	0.0000	0.6556	0.6556	0.0000
150	0.3057	0.3057	0.0000	0.2887	0.2887	0.0000
200	0.1945	0.1945	0.0000	0.1879	0.1879	0.0000
300	0.1254	0.1254	0.0000	0.1242	0.1242	0.0000
400	0.1010	0.1010	0.0000	0.1011	0.1011	0.0000
500	0.0879	0.0880	0.0000	0.0885	0.0885	0.0000
600	0.0793	0.0793	0.0000	0.0800	0.0800	0.0000
800	0.0680	0.0680	0.0000	0.0688	0.0688	0.0000
1000	0.0604	0.0604	0.0000	0.0612	0.0612	0.0000
1500	0.0490	0.0490	0.0000	0.0496	0.0496	0.0000
2000	0.0428	0.0428	0.0000	0.0433	0.0433	0.0000
3000	0.0365	0.0365	0.0000	0.0366	0.0367	0.0000
4000	0.0335	0.0335	0.0000	0.0334	0.0334	0.0000
5000	0.0319	0.0319	0.0000	0.0316	0.0316	0.0000
6000	0.0311	0.0311	0.0000	0.0306	0.0306	0.0000
8000	0.0306	0.0306	0.0000	0.0297	0.0297	0.0000
10,000	0.0307	0.0307	0.0000	0.0296	0.0296	0.0000
15,000	0.0319	0.0319	0.0000	0.0304	0.0304	0.0000
**Photon energy (keV)**	**S1**			**TPU**		
	**Phy-X** **(cm^2^/g)**	**WinXCom** **(cm^2^/g)**	**Δ (%)**	**Phy-X** **(cm^2^/g)**	**WinXCom** **(cm^2^/g)**	**Δ (%)**
15	36.1943	36.2000	−0.0057	1.1003	1.1000	0.0003
20	16.6824	16.6800	0.0024	0.5694	0.5694	0.0000
30	11.1289	11.1300	−0.0011	0.3009	0.3009	0.0000
40	5.2087	5.2100	−0.0013	0.2327	0.2327	0.0000
50	2.8799	2.8800	−0.0001	0.2051	0.2051	0.0000
60	1.7902	1.7910	−0.0008	0.1901	0.1901	0.0000
80	0.8748	0.8749	−0.0001	0.1729	0.1729	0.0000
100	0.5277	0.5278	−0.0001	0.1620	0.1620	0.0000
150	0.2511	0.2511	0.0000	0.1437	0.1438	−0.0001
200	0.1731	0.1731	0.0000	0.1311	0.1311	0.0000
300	0.1214	0.1214	0.0000	0.1137	0.1137	0.0000
400	0.1012	0.1012	0.0000	0.1017	0.1017	0.0000
500	0.0896	0.0896	0.0000	0.0929	0.0929	0.0000
600	0.0815	0.0815	0.0000	0.0859	0.0859	0.0000
800	0.0705	0.0705	0.0000	0.0754	0.0754	0.0000
1000	0.0629	0.0629	0.0000	0.0678	0.0678	0.0000
1500	0.0510	0.0510	0.0000	0.0552	0.0552	0.0000
2000	0.0443	0.0443	0.0000	0.0473	0.0473	0.0000
3000	0.0370	0.0370	0.0000	0.0380	0.0380	0.0000
4000	0.0332	0.0332	0.0000	0.0325	0.0325	0.0000
5000	0.0309	0.0309	0.0000	0.0288	0.0289	0.0000
6000	0.0295	0.0295	0.0000	0.0263	0.0263	0.0000
8000	0.0280	0.0280	0.0000	0.0229	0.0229	0.0000
10,000	0.0274	0.0274	0.0000	0.0209	0.0209	0.0000
15,000	0.0273	0.0273	0.0000	0.0181	0.0181	0.0000

## Data Availability

Data will be made available on request.

## References

[1] Yun J., Hou J., Jang W., Kim S.Y., Byun H. (2021). Electrospun Tungsten-Polyurethane Composite Nanofiber Mats for Medical Radiation-Shielding Applications. ChemNanoMat.

[2] Yu L., Yap P.L., Santos A.M.C., Tran D.N.H., Losic D. (2023). Lightweight polyester fabric with elastomeric bismuth titanate composite for high-performing lead-free X-ray shielding. Radiat. Phys. Chem..

[3] Hashemi S.A., Mousavi S.M., Faghihi R., Arjmand M., Sina S., Amani A.M. (2018). Lead oxide-decorated graphene oxide/epoxy composite towards X-Ray radiation shielding. Radiat. Phys. Chem..

[4] Akkurt I., Alomari A., Imamoglu M.Y., Ekmekçi I. (2023). Medical radiation shielding in terms of effective atomic numbers and electron densities of some glasses. Radiat. Phys. Chem..

[5] Okafor C.E., Okonkwo U.C., Okokpujie I.P. (2021). Trends in reinforced composite design for ionizing radiation shielding applications: A review. J. Mater. Sci..

[6] Hosseini S.H., Askari M., Ezzati S.N. (2014). X-ray attenuating nanocomposite based on polyaniline using Pb nanoparticles. Synth. Met..

[7] AbuAlRoos N.J., Baharul Amin N.A., Zainon R. (2019). Conventional and new lead-free radiation shielding materials for radiation protection in nuclear medicine: A review. Radiat. Phys. Chem..

[8] Jamal AbuAlRoos N., Azman M.N., Baharul Amin N.A., Zainon R. (2020). Tungsten-based material as promising new lead-free gamma radiation shielding material in nuclear medicine. Phys. Med..

[9] Seenappa L., Manjunatha H.C., Chandrika B.M., Sridhar K.N., Hanumantharayappa C. (2018). Gamma, X-ray and neutron interaction parameters of Mg–Gd–Y–Zn–Zr alloys. Radiat. Phys. Chem..

[10] Rani N., Vermani Y.K., Singh T. (2020). Gamma radiation shielding properties of some Bi-Sn-Zn alloys. J. Radiol. Prot..

[11] Rammah Y.S. (2020). Influence of Ag2O insertion on alpha, proton and γ-rays safety features of TeO2.ZnO.Na2O glasses: Potential use for nuclear medicine applications. Ceram. Int..

[12] Prabhu S., Bubbly S.G., Gudennavar S.B. (2022). X-Ray and γ-Ray Shielding Efficiency of Polymer Composites: Choice of Fillers, Effect of Loading and Filler Size, Photon Energy and Multifunctionality. Polym. Rev..

[13] More C.V., Alsayed Z., Badawi M.S., Thabet A.A., Pawar P.P. (2021). Polymeric composite materials for radiation shielding: A review. Environ. Chem. Lett..

[14] Kim S., Ahn Y., Song S.H., Lee D. (2022). Tungsten nanoparticle anchoring on boron nitride nanosheet-based polymer nanocomposites for complex radiation shielding. Compos. Sci. Technol..

[15] Wang H., Zhang H., Su Y., Liu T., Yu H., Yang Y., Li X., Guo B. (2015). Preparation and radiation shielding properties of Gd2O3/PEEK composites. Polym. Compos..

[16] Bhardwaj N., Kundu S.C. (2010). Electrospinning: A fascinating fiber fabrication technique. Biotechnol. Adv..

[17] Eatemadi A., Daraee H., Zarghami N., Melat Yar H., Akbarzadeh A. (2016). Nanofiber: Synthesis and biomedical applications. Artif. Cells Nanomed. Biotechnol..

[18] Massoumi B., Massoumi R., Aali N., Jaymand M. (2016). Novel nanostructured star-shaped polythiophene, and its electrospun nanofibers with gelatin. J. Polym. Res..

[19] Noor Azman N.Z., Wan Mohamed W.F.I., Ramli R.M. (2022). Synthesis and characterization of electrospun n-ZnO/n-Bi2O3/epoxy-PVA nanofiber mat for low X-ray energy shielding application. Radiat. Phys. Chem..

[20] Hazlan M.H., Jamil M., Ramli R.M., Noor Azman N.Z. (2018). X-ray attenuation characterisation of electrospun Bi2O3/PVA and WO3/PVA nanofibre mats as potential X-ray shielding materials. Appl. Phys. A.

[21] Ye D., Peng Z., Liu J., Huang Y. (2022). Self-Limited ultraviolet laser sintering of liquid metal particles for μm-Thick flexible electronics devices. Mater. Des..

[22] Liu H., Xin Y., Bisoyi H.K., Peng Y., Zhang J., Li Q. (2021). Stimuli-Driven Insulator-Conductor Transition in a Flexible Polymer Composite Enabled by Biphasic Liquid Metal. Adv. Mater..

[23] Deng Y., Liu J. (2015). Liquid Metal Based Stretchable Radiation-Shielding Film. J. Med. Devices.

[24] Lou P., Teng X., Jia Q., Wang Y., Zhang L. (2019). Preparation and structure of rare earth/thermoplastic polyurethane fiber for X-ray shielding. J. Appl. Polym. Sci..

[25] Wu J., Hu J., Wang K., Zhai Y., Wang Z., Feng Y., Fan H., Wang K., Duan Y. (2023). Flexible stretchable low-energy X-ray (30–80 keV) radiation shielding material: Low-melting-point Ga(1)In(1)Sn(7)Bi(1) alloy/thermoplastic polyurethane composite. Appl. Radiat. Isot..

[26] Wang K., Hu J., Chen T., Tang J., Zhai Y., Feng Y., Zhao Z., Fan H., Wang K. (2021). Radiation shielding properties of flexible liquid metal-GaIn alloy. Prog. Nucl. Energy.

[27] Agar O., Tekin H.O., Sayyed M.I., Korkmaz M.E., Culfa O., Ertugay C. (2019). Experimental investigation of photon attenuation behaviors for concretes including natural perlite mineral. Results Phys..

[28] Singh T., Kaur P., Singh P.S. (2007). A study of photon interaction parameters in some commonly used solvents. J. Radiol. Prot..

[29] Han I., Demir L. (2009). Determination of mass attenuation coefficients, effective atomic and electron numbers for Cr, Fe and Ni alloys at different energies. Nucl. Instrum. Methods Phys. Res. Sect. B Beam Interact. Mater. At..

[30] Rashidi M., Rezaei A., Bijari S., Jaymand M., Samadian H., Arkan E., Zahabi S.S., Hosseini M. (2021). Microfibers nanocomposite based on polyacrylonitrile fibers/bismuth oxide nanoparticles as X-ray shielding material. J. Appl. Polym. Sci..

[31] Li X., Li M., Zong L., Wu X., You J., Du P., Li C. (2018). Liquid Metal Droplets Wrapped with Polysaccharide Microgel as Biocompatible Aqueous Ink for Flexible Conductive Devices. Adv. Funct. Mater..

[32] He J.H., Liu Y., Xu L. (2013). Apparatus for preparing electrospun nanofibres: A comparative review. Mater. Sci. Technol..

[33] Haider A., Haider S., Kang I.-K. (2018). A comprehensive review summarizing the effect of electrospinning parameters and potential applications of nanofibers in biomedical and biotechnology. Arab. J. Chem..

[34] Lasprilla-Botero J., Álvarez-Láinez M., Lagaron J.M. (2018). The influence of electrospinning parameters and solvent selection on the morphology and diameter of polyimide nanofibers. Mater. Today Commun..

[35] Ji Z., Han Y., Yan L., Jian Z., Hongyu S., Ning M. (2018). Estimation of viscosity and phase inversion point for oil-water mixture. Energy Procedia.

[36] Lin Y., Genzer J., Dickey M.D. (2020). Attributes, Fabrication, and Applications of Gallium-Based Liquid Metal Particles. Adv. Sci..

[37] Tang Q., Gao K. (2017). Structure analysis of polyether-based thermoplastic polyurethane elastomers by FTIR, 1H NMR and 13C NMR. Int. J. Polym. Anal. Charact..

[38] Tekin H.O., Kilicoglu O. (2020). The influence of gallium (Ga) additive on nuclear radiation shielding effectiveness of Pd/Mn binary alloys. J. Alloys Compd..

[39] Kaur T., Sharma J., Singh T. (2019). Review on scope of metallic alloys in gamma rays shield designing. Prog. Nucl. Energy.

[40] Mahmoud K.A., Tashlykov O.L., Sayyed M.I., Kavaz E. (2020). The role of cadmium oxides in the enhancement of radiation shielding capacities for alkali borate glasses. Ceram. Int..

